# Case of Extra-Uterine Pregnancy, Continuing Four Years; Fœtus Removed by Gastrotomy; Recovery

**Published:** 1861-09

**Authors:** E. P. Cook

**Affiliations:** Mendota, Illinois


ARTICLE II.

### CASE OF EXTRA-UTERINE PREGNANCY.

### CONTINUING FOUR YEARS.---FŒTUS REMOVED BY GASTROTOMY---RECOVERY.

By E. P. COOK, M. D., of Mendota, Illinois.

Mrs. Getchell, the subject of the following report, below the medium height; thin, never more than ninety-five pounds; of a delicate organization; highly excitable, nervous temperament; age twenty-eight years. Was first called to see her February 27th, 1857. Supposed herself to be in her third pregnancy; had menstruated last, October 27th, 1856. Had had symptoms similar to those occurring during her previous pregnancies—cessation of menses, morning sickness, swelling, soreness and secretion in breasts.

The day previous to my being called, had exerted herself rather more than usual, and during the night had pretty severe periodical pains in lower part of abdomen, with some hæmorrhage. This becoming worse, I was called. From the history of her case and present condition, I was at once led to think that she was pregnant, and unless uterine action were immediately arrested an abortion would be the result. Resorted to the ordinary treatment in such cases, and in the course of forty-eight hours the flowing had ceased, but still some pain, which continued more or less for several days. Rest was advised, and the horizontal posture as much as possible. At the expiration of the fourth month she thought she felt motion or the quickening, and declared that she felt more or less movement continuously, as usual in pregnancy, which was also affirmed by her husband; the movements being quite distinct, though not so strong and frequently perceptible as they had been in her previous pregnancies.

Up to July 26th, which would make nine months, the abdomen gradually and uniformly enlarged, and at the expiration of that time was about the usual size of a woman of her development at full term. We would observe here that the lady was not seen by us during the interval between the period of quickening and after the expiration of the full term. From her statements we learned that she suffered rather more than usual the common inconveniences to which the pregnant female is incident; but there was no material deviation from her usual general health, nor any circumstances that excited apprehension of a serious character in her own mind as to her condition and the final result.

July 26th.—In the absence of the writer, his father, Dr. W. L. Cook, was called to see her, as she supposed herself to be in labor. Had considerable show, and frequent severe pains, from the irregularity and character of which, he soon became satisfied that she was not in actual labor, and gave her an anodyne; the pains soon ceased. She ran along then without change in size, feeling no movement, but suffering from pains (which she compared to the pains of the first stage of natural labor) occasionally, but most severely at the expiration of every month. The general health improved, and she was again able to take charge of her domestic affairs.

In September, 1857, the catamenia returned, and she continued perfectly regular up to the time of operating. The case did not then receive any special attention from me until January 31st, 1858. Mr. and Mrs. G. becoming still more uneasy in reference to her condition, I was requested to call, and if possible, to give a positive opinion in reference to her case, and the better course to be pursued. I would here remark that up to the expiration of full term, I had scarcely any suspicions that the case was not one of ordinary pregnancy, From its continuance, however, without any material change, the case became one of peculiar interest. Condition January 31st, 1858: Looked very well, countenance cheerful, in usual flesh, appetite good, bowels regular, menstruates every month, does not suffer as much at that period as she formerly did, occasionally has some pain in the lower part of her abdomen—more at night. No change in size since time at which she was last seen; enlargement of abdomen uniform and elastic; could get the outlines of the tumor distinctly, resembling very accurately the form of the impregnated uterus at full term. By carefully comparing the enlargement to either side of the mesian line, it was evident that the greater bulk of the tumor lay to the left side, but the difference not very marked. No unusual sensitiveness on either pressure or palpation. Dull sound on percussion; cannot discover any fluctuation; change of position of body does not change position of tumor; careful examinations per vagina and rectum, did not reveal anything worth noting, except the encroachment of the tumor upon the superior border of the pelvis, and forcing the uterus, etc., low in its cavity; could not by any proper means change position or elevate it in the least. Palpation or any manipulation externally over the abdomen, would not communicate to index finger in vagina a sense of fluctuation.

February 16th, 1859.—No perceptible change in her condition in any respect since last notings; would see her occasionally, and learn that she "was quite smart," as well as when in her best health previously. For the last two weeks has been suffering considerably with pain through the lower part of her abdomen—left side—and for several nights extremely, with pains resembling first stage labor pains. Re-examined the case carefully, nothing discovered worth the noting; to take anodynes *pro re nata.* Her case ran along thus during the spring and summer of 1859, without materially changing, excepting the increase in her suffering—almost constant pain, and the degree of mental anxiety about her condition, producing a gradual loss of flesh and physical strength.

In September 1859, through the influence of friends and others, Mrs. G. was prevailed upon to visit Wisconsin, with the assurance that *a radical cure* of her case would then be effected.

She returned to Mendota, Feb. 1, 1860, shortly after which she was again seen by me, and from her I learned that her condition continued about the same as at the time of leaving, until about Dec. 1, when it became evident that her case was undergoing some change,—her abdomen becoming fuller, and at an examination made by two or three physicians, fluctuation was found to be quite distinct. Being satisfied of the presence of fluid in the abdominal cavity, tapping was done, and from three to three and one-half gallons of fluid of a sero-purulent character evacuated.

She recovered fully from the tapping in a few days, and was so much relieved, temporarily, as to be flattered with the hope that she was *radically cured.*

The operator informed her that, on introducing a probe through the canula into the cavity, that it came into contact with *bones*, and that *hair* and small pieces of cartilage were found in the fluid evacuated.

Condition, Feb. 3, 1860.—General appearance better than when last seen; is very cheerful; thinks she is almost well; abdomen much less distended, but circumference over the most prominent part of the tumor only three inches less than it was eight months since, but the enlargement does not extend so high in the abdomen; fluctuation quite distinct; on palpation to the left of the mesian line, just below the umbilicus, a sensation is communicated to the fingers, which I can readily think might be produced by some solid body floating or lying in a sac containing fluid.

It was very evident that the sac would soon be again distended by a re-accumulation of fluid, and candor required us to so inform the patient. Uniformly, but slowly, the accumulation of fluid progressed, and with its increase, she again began to suffer, her pain increasing with the increased distension; loss of rest and appetite, with emaciation resulting, and telling rapidly on her delicate organization. By June 3rd, her condition was such as to make it important, that either the fluid be re-evacuated, or an operation instituted, which would terminate the case fully and finally in either a perfect recovery or death.

The patient and friends preferring that she should be tapped rather than take the risk of gastrotomy—that being presented to them as the only alternative beyond the temporary relief that might be afforded by repeated tappings. They, however, decided that it should be left to my judgment, and those whom I might associate with me as assistants and advisers.

All necessary preparations were made, and on June 3, in the presence of, and with the assistance of the neighboring physicians, a trocar was introduced in the mesian line of the abdomen, about two inches below the umbilicus, and two and one-half gallons of sero-purulent matter evacuated. A sound was then introduced through the canula into the sac, and with all the care and perseverance thought advisable, the sound could not be made to reach any bone or bones. Foiled in this, we hastily determined to defer any further surgical procedure. Removed the canula, covering the trifling wound made in the integument, with lint and adhesive straps, over which put compress and bandage.

In a few days she was again up, very comfortable, taking food generally, and her system recuperating rapidly. But a few weeks of rapid secretion by the sac, placed her case again where it had been twice previously. To abbreviate, we will state that it was necessary to tap again:—Aug. 7, Sept. 5, Sept. 30. At the third tapping, tincture of iodine was injected into the cavity, with no effect, except to render convalesence sure. It will be observed that the interval between the several tappings became less as the case advanced; this is explained by the fact, that secretion went on much more rapidly after each tapping, and the temporary relief to her sufferings was much shorter—a less degree of distension of the sac being required to produce it again, yet, at no time was less than two gallons of fluid removed. By the first of October, her almost constant suffering, loss of rest, and the excessive drain on her system, she was so far succumbing, as to make it very evident that life would soon be terminated. She now became very anxious to avail herself of the only hope for relief that could be offered, and though the formidable nature of gastrotomy was candidly presented to her, she still insisted that it should be performed, at the same time saying, with all the confidence of which poor suffering humanity is sometimes capable, that it would certainly cure her, *that she would live and not die.* It then clearly became my duty to give the patient the benefit of an operation for the removal of the cause, and not simply the effects as had only been done previously.

A surgeon's course, under such circumstances, becomes a moral question of no small importance. With the conviction that a patient should not be allowed to go through a life of suffering to inevitable death, when there is a possibility that an operation may result in a restoration to health, it is clearly his duty to do all he can for the poor creature reposing confidence in him. This matter was fully considered and acted upon in this case. Having determined on the operation, surrounding conditions and the state of the patient were made as favorable as possible, waiting until she should recover from the last tapping, and the sac had time to be pretty well filled again. Accordingly, every arrangement having been made, on the afternoon of October 14, in the presence and with the kind assistance of Drs. Jos. W. & F. H. Edwards, Corbus, Gould, and Dr. W. J. Cook, the operation was performed.

I had determined, with the advice of the physicians named, to make the abdominal incision, evacuate the fluid, remove the fœtus, if such we found, and attempt the removal of a part or the entire sac, if its attachments were not such as to render it impossible or inexpedient.

The atmosphere of the room was brought to a temperature of about 70°, and kept moist by the evaporation of water from kettles on the stove. The patient was placed on a bed in the middle of the room, with her shoulders well raised, and a sheet applied as a diaper. She was then put under the influence of chloroform, and I made an incision in the *linea alba* about five inches in length (being just below the umbilicus, and extending toward the pubis) down to the peritoneum. The sac was then punctured, and from two to three gallons of fluid of the same character as that previously removed, evacuated, emptying the sac completely. The adhesions of the sac to the peritoneum at the place of the incision were easily broken down, and the hand passed into the cavity of the abdomen above the sac, the greater omentum was found, partially adherent to the superior surface of the sac; and as the hand was passed further down posteriorly and laterally, the sac was found so firmly adherent in the left iliac fossa, and over the lower lumbar vertebra, and also, partially in the right illiac fossa, as to render it impossible to remove it without using the scalpel, and dissecting it out, which we had determined, previously, not to do, of the propriety of which determination we were more fully convinced than ever. The incision in the sac was then enlarged, and the hand introduced, and the fœtus (weighing five pounds) removed without any difficulty; the fœtus was entire and in a perfect state of preservation, excepting scalp and bones of the cranium; the softening of the scalp and separation of the superior bones of the cranium was such, that on lifting out the fœtus, they remained in the bottom of the sac; they were carefully removed, but no trace of either cord or placenta were found. The cavities of the sac and abdomen were then carefully sponged out, (there having been in all, not more than two and a half or three ounces of blood lost,) no arterial branches requiring the ligature. After careful sponging, the incision in the sac and abdomen were brought together conjointly by passing large steel needles through at each extremity of the incisions in both sac and abdomen, the needle entering the integument about a half-inch from margin of wound, passing obliquely through the abdominal wall, taking up both lips of the sac, (including peritoneum,) and emerging through opposite edge of incision to that at which it had entered. Around each needle were thrown several turns of the twisted suture. Six sutures were then put in, three on each side, which brought neatly together the free edges of wounds in sac and abdomen, leaving an opening from the surface two inches long, communicating with the cavity of the sac. By thus dressing it, the peritoneal coverings of abdominal walls and of the sac at the margin of the incisions were brought firmly and perfectly in contact, everting and bringing the lips of the sac to the surface, isolating perfectly the cavity of the sac from that of the abdomen, and leaving a fistulous opening communicating freely with it. The surface was then dressed with lint and several strips of adhesive plaster; over all a light compress and bandage, and water dressing; operation completed at 2½ o'clock p. m. By the time the dressing was completed, had rallied partially. Pulse 100, very feeble; complaining frequently of a severe burning pain at the seat of the incisions. Gave her immediately after the stupefaction from the chloroform had passed off, 1 gr. sulph. morphine, each hour to take ½ gr. At 5 o'clock, pulse 110, more volume, lower extremities cold, vomited freely shortly after the operation was completed, not since. Nine o'clock p. m. Pulse 110, general surface of body and limbs warm; reaction complete; suffers from frequent and severe pains which she compares to *after-pains*; has vomited again; continued the opiate freely. Twelve o'clock m.—pulse same; has vomited again; is more comfortable, but still those pains return occasionally; very little sleep; has evacuated 4℥ urine; *via naturalis.* Eight o'clock a. m.—Since midnight has slept nearly three hours; vomited twice; evacuated ½ pint urine; pulse 95, weak; expresses herself as feeling very weak and faint; general surface normal temperature; but little pain; takes beef tea, ice, brandy frequently in small quantities. Eleven o'clock.—Continues to vomit occasionally; sleeps considerably; apparently never prostrated; pulse 115; continue to give opiates at intervals of from two to six hours, and in quantities, varying from ¼ to 1 gr. sul. morphine, designing to keep her system under their decided influence. It will be remembered that from habit, her system tolerates quantities that, under ordinary circumstances, would be enormous. Gave her hydrocyanic acid, without any apparent effect in controlling the vomiting.

One o'clock p. m., 15th Oct.—No material change in any particular; vomits frequently; left Dr. Jas. Edwards in charge of the case.

Seven o'clock p. m.—Has vomited three or four times since 1 o'clock, most of the time resting very comfortably; describes her sleep as being very refreshing; has evacuated urine again; pulse 125, full, skin hot, free from pain. Up to this time there has been no tympanitis or tenderness of her abdomen.

October 16, 7 o'clock a. m.—Pulse 115, not so full; less heat of surface, and less thirst; rested well during the night; no more vomiting; complains of an itching sensation over whole person; dressed the wound, looks well, beginning to suppurate, opening into sac, patulous, whole wound contracted to less than three inches in length.

Seven o'clock p. m.—No material change; pulse not so frequent, has ranged during the day from 95 to 110; sleeps considerably.

4th day, Oct. 17, 8 o'clock a. m.—General condition the same; rested pretty well last night; pulse from 120 to 105 this morning; evacuates (every three to four hours,) with but little difficulty, from 4 oz. to ½ pt. urine; takes beef-tea, rice-water, tea, crackers, etc., in small quantities, frequently.

5th day, Oct. 18.—For the last three days there has been a decided degree of febrile excitement, (with some delirium,) evidently the result of some inflammatory action at the seat of the operation, though there is no evidence as yet, of the occurrence of general peritonitis; abdomen but slightly tympanitic or tender; dressed the wound this morning—looks well; suppurating freely; discharging some from the sac. Takes a pill sulph. morphine, ½ gr. every six hours.

6th day, Oct. 19.—Rested well last night; pulse 105; wound discharging freely; is very tired of beef-tea; discontinue it, and substitute the broth strong; the mind has become very child-like; some delirium; debility more marked; pulse 130.

7th day, Oct. 20.—Pulse 110; debility more marked than ever; wound and sac discharging very freely; fetor very unpleasant; abdomen more tympanitic and tender, particularly to the right of the mesian line; bowels were evacuated freely by enemas of beef broth. For two days, has suffered much from an apthous affection of the mouth and throat; put her to-day on the use of wine-whey; chlor. potassa and tannin as a gargle for her mouth; to take a pill every four hours, sulph. quinine 1 gr., pulv. opium, 1½ gr.; to apply over dressing to wound, cloths saturated with solution chlor. lime.

8th day, Oct. 21.—Complains less of pain in right side of abdomen; less tympanitis; pulse 100; mouth and throat very sore; the condition of mouth and throat is identical with that of stomatitis materni; this morning removed the pins; union firm and perfect; length of incision not healed, 2 in; fistulous opening into sac, ¾ in.

9th day.—Pulse ranging from 90 to 95; wound suppurating freely; not so much fetor; but little tympanitis; right side of abdomen very tender, and complains much of a dragging pain there; exacerbations of the amount of arterial excitement occur during the afternoons and fore part of nights.

10th day, Oct. 23.—Pulse 100; more cheerful; thinks she will recover; takes more nourishment; oyster broth added to her bill of fare; rested well last night; right side of abdomen less painful and tender, but more tympanitic than was yesterday; secretion of urine and its evacuation the same as previously noted; is stronger, can help herself more. Since removing the needles, the wound has gaped considerably; fistulous opening one inch long—communicating freely with interior of sac, from which there is a free discharge of fœtid purulent matter; surface of wound mostly granulating, with a very little sloughing at the lower extremity; dress with lint and adhesive straps, over which apply light compress and flannel bandage, over all apply cloths frequently wrung out of chlor. lime water. Directed an enema of beef broth, the same as was done three days since.

11th day, Oct. 24, 8 o'clock p. m.—Pulse 120; restless; more delirium than has ever been; extremities cool; abdomen less tender, more tympanitic; takes less nourishment; mouth and throat the same; stimulants to be increased; cold to head, warmth to extremities.

12th day, Oct. 25.—Rested better last night than was expected; bowels evacuated freely, after repeated injections; the difficulty of the mouth and throat is increasing; takes alternately with the pills of opium and quinine, comp. tinct. bark, with chlor. potassa in solution.

13th day, Oct. 26.—Mouth very sore; deglutition difficult and painful; complains of a burning sensation and feeling of constriction along the course of the œsophagus, and also to some extent in the epigastric region; has vomited several times to-day. The diseased state of mucous membrane is evidently extending itself down along the course of the œsophagus, and invading the stomach. The mucous membrane of the mouth and throat is peeling off extensively, leaving the denuded and red surface exposed, which is very sensitive. Pulse good and only 100; general condition aside from the difficulty referred to, good; temperature natural, but skin has always been dry; the wound presents a good appearance; edge granulating slowly, and contracting in every dimension; free secretion of semi–purulent matter from sac, but little odor; the sac still continues to a limited extent, to imitate its old secretory function.

14th day, Oct. 27.—Bowels evacuated naturally, twice to-day; continues to take powders, quinine et morphine every six hours; as a local application to mouth and throat, chlor. potassa in infusion of hydrastis canadensis; wound doing well.

15th day, Oct 28.—If it were not for the severity of the affection of her mouth, throat and œsophagus, her condition in every other respect would be good. The wound is looking well. Deep in the wound lie the lips of the sac. It has been gradually retracting, until now it is seen at a depth of nearly one inch from the surface of the abdomen, leaving a cupped depression at the seat of the original incisions.

Deglutition very difficult and painful; complains most of a severe burning pain over the region of the middle third of the œsophagus. In all probability the surface is there ulcerated. It is only with considerable effort that she can open her mouth to admit light enough to examine the fauces. The epithelial layer of mucous membrane peels off in large flakes, leaving a red and inflamed base, upon which a rapid exudation occurs, which becomes organized into a pseudo-membranous formation, to be rapidly thrown off, to be succeeded by the same again. The only ulcerated surface that can be seen, is at the angles of the mouth. A slight diarrhœa now existing; pulse ranging from 120 to 130—feeble; is restless; mind wandering; has been harrassed by a very annoying hacking cough for two or three days; the quinine and morphine to be continued; the potassa discontinued, substituting mur. tr. iron, both for its constitutional and local effects, 20 gtt. to be diluted sufficiently, and given every four hours; to take all the concentrated nourishment possible.

16th day, Oct. 29.—Is more comfortable; complains less of her throat; pulse from 110 to 120; she thinks that the iron is helping her very much, and it unquestionably is having a good effect; the mouth and throat is less sensitive; the exudation is less in amount, and is thrown off much more easily.

18th day, Oct. 31.—Appetite improving; mouth and throat better; deglutition less painful; in the evening, was sitting up in bed, taking a cup of tea and a soft boiled egg; the abdomen has been in the least tympanitic for three or four days, very little tenderness remaining; is very cheerful, and thinks she will certainly get well; bowels are evacuated *via naturalis* every day; no difficulty in urinating.

Nov. 1, 19th to 25th day,—since the operation.—Mouth and throat have continued to improve ever since she began the use of the tr. ferri chlor.; generally rests quite comfortably, and takes nourishment freely. For several days, though the wound has in other respects been doing finely, yet over its entire free surface, and extending down into the cavity as far as can be seen, there has been a pseudo-membranous exudation found, resembling precisely the appearance of the mouth and throat, as far as can be seen. She has complained very much of the wound's smarting and burning, causing so much nervous excitement, as to make it necessary to return to the use of the sulph. morphine, which had been discontinued for several days.

25th to 30th day, Nov. 6 to 11.—For the last six days her condition has not been so good as it was previously, and she is evidently losing, instead of gaining; debility more marked, and is becoming more emaciated; takes less nourishment; rests about the same; is desponding, and begins to think at times that she cannot recover; mouth still continues sore, but is much better; no difficulty in swallowing; skin generally hot and dry; suppuration continued quite free from the sac; pus of a normal character; some fœtor; external wound gradually contracting, but opening into sac is larger, 1½ in. long. A probe can be passed down three inches towards the right iliac fossa, and not at all to left of mesian line. At this stage of her case, the prospects are fearfully against her final recovery; she continued, however, as generous a use as possible of concentrated nourishment,—stimulants, opiates, etc.

Nov. 16.—Since last notings there has been a gradual improvement in her condition in every respect: takes food freely; rests better at night; does not complain of the wound; is stronger; bowels in good condition; external wound growing smaller slowly; cavity of sac suppurating freely; pus very thick; probe passes to a depth of 2½ in. now. Have discontinued all medicines now, except the occasional use of il opii et fluid ext. valerian.

From this on she slowly, but uniformly, improved; becoming stronger, more comfortable, etc. The discharge from the sac decreasing in amount gradually, and by March 1, 1861, she was again able to take charge of her domestic duties.

May 1, 1861.—Her health is as good as it ever was; in her usual flesh; enjoys life and its pleasures as much as she ever did. In reference to the sac now remaining in her abdomen, we would observe that there still remains a very small fistulous opening at the bottom of the depression at the seat of the original incisions, through which a small probe can be passed to the depth of an inch, and that from it there continues to exude a trifling amount of pus. Through the walls of the abdomen the sac may be easily felt, about the size of the closed fist, firmly fixed just below the umbilicus, extending down to the left iliac fossa, the greater bulk to the left of the mesian line.

---

Remarks.—It will be a matter of interest to refer briefly, yet more at length than has been done, to some of the features of this interesting case. It may appear very clear, since the history of the case is now perfect, that its *diagnosis* was an easy one at any stage after the time at which I was first called, yet such was not the case. It was with difficulty, and only after a protracted observation of the case, that I became fully satisfied as to its nature. It is true, that up to the expiration of the period of natural pregnancy, I had no suspicions that the case was not one of ordinary pregnancy; and still, I had considered the case as such, simply from the circumstances attending it at the time when I was first called, and the statements of the lady herself in reference to the movements of the fœtus subsequently. After the expiration of the nine months, and the case remaining without change, it continued to become increasingly interesting. For a while I doubted the fact of there ever having been fœtal movement, not from any want of confidence in the lady's veracity, but thought she might have been deceived herself. We are well aware that, occasionally, in cases of hydatinous or sarcomatous moles, and under other peculiar circumstances, patients have construed certain sensations felt, to be the result of fœtal movements. Yet, after all, there could be no diagnosis of the case made to which there were so few objections as that of considering it as one of *extra-uterine pregnancy*—the evidence confirmatory of such diagnosis accumulating as the case advanced.

Some of the features of this case are of such interest, and one at least peculiarly so, as to demand more than a passing notice (yet, a desire to be brief shall prevent us from reviewing them minutely). We here refer to the fact that after the fœtus had been carried in its *provisional uterus*, or sac propper, without any change after the expiration of nine months, until more than three years had passed, then the lining membrane of the sac took upon itself a secretive function, and gave rise to a state of things resembling very much a case of ovarian dropsy; and repeated tappings were necessary. We have not been able, in all our researches into the history of cases of extra-uterine pregnancy, to find a parallel case in this particular. At the time the embryo secured its primitive connection where it had been arrested and developed in its progress from the ovary to the uterus, indicated by the occurrence of symptoms identical with those occurring in connection with an abortion, there was no sensation of tearing or giving way of any of the structures in the abdomen or pelvis. At this time, undoubtedly, the primative cysta of the ovulum was ruptured, without the severing of any large vessel; the internal hemorrhage very moderate; a maternal relation still sustained to a degree compatible with the future development of the ovum; ovum lodging in the peritoneal cavity, giving rise to slight peritoneal irritation, yet within such limits as not to cause general and severe inflammation thereof.

At the time the rupture occurred, by sympathy, the contracted fibres of the uterus were thrown into action, causing pain, and by the same, detaching the decidua, whence the blood had its origin.

At the expiration of the normal term of pregnancy, the fœtus ceased to live, after which, for one year it was carried, the woman enjoying as good health as usual. During the next year and a half, we see the endeavors of the female economy to get rid of its burden. In December, 1859, this is first observed in the amount of abdominal distension, and by the latter part of January, 1860, it becomes very evident that this was owing to the presence of fluid, for which tapping was done. From that time until date of the final operation,—at five different tappings, and inclusive of the amount of fluid removed at the time of operating, there was from 16 to 18 gallons of fluid removed.

The occurrence of the pains, shortly after the completion of the operation, and their persistence for several days is explained by the fact of the muscular character of the sac, and they were unquestionably the result of its periodic contraction, as occurs with the uterus. It is an interesting question as to what might be the consequence of the occurrence of a future pregnancy. The position of the sac and its extensive and firm attachments, are almost, if not entirely, such as to make impossible the rising of the impregnated uterus out of the pelvis, as it develops, and yet all are aware of the surprising distensibility of the female organism. The favorable result in this case is, no doubt, attributable to a variety of circumstances, not the least of which was the determination and confidence on the part of the patient that she should recover; and the possession of an organism with such vital tenacity, that nothing short of *articulo mortis* itself would be sufficient to forbid hope. And yet the successful result was in consequence of allowing the sac to remain without forcibly attempting its removal, and the immediate and perfect union of lips of sac to abdominal walls, and establishing a fistulous opening, communicating externally.

I would here express my obligations to Dr. Jas. W. Edwards of this place, for the kind and valuable assistance rendered me during the progress of this case · And also to Dr. Jas. N. Niglas, of Peoria, and Dr. N. S. Davis, of Chicago, for the interest they have taken in its progress, and valuable advice given me.

